# Case 3/2016 - 36-Year-Old Man with Anomalous Origin of the Right Coronary Artery in the Left Sinus of Valsalva and Interarterial Course

**DOI:** 10.5935/abc.20160051

**Published:** 2016-04

**Authors:** Edmar Atik, Roberto Kalil Filho, Marcelo Jatene

**Affiliations:** Hospital Sírio Libanês de São Paulo, São Paulo, SP - Brazil

**Keywords:** Cardiac Surgical Procedures, Coronary Vessel Anomalies / surgery, Heart Defects, Congenital, Sinus of Valsalva, Cardiac Catheterization

**Clinical data:** Three months ago, after physical effort, the patient had four episodes of paleness, excessive sweating and fatigue, relieved when lying down in supine position. In addition, the patient had pain of 2-hour duration in the left hemithorax at normal activity level a few days ago.

Physical examination showed good general condition, not pale, anicteric, eupneic, normal pulse, and no palpable pulse in the suprasternal notch was detected. Body weight was 85 kg, height 170 cm, BP 120/70 mmHg, HR 75 bpm. No abnormalities in the precordium, normophonetic heart sounds, and no heart murmurs. No changes in the lungs or abdomen were detected.

## Complementary tests

**Electrocardiogram** showed sinus rhythm, no signs of atrial or ventricular overload and no changes in the ventricular repolarization. AP = +50º, AQRS = +40º, AT = +60º

**Figure 1:**
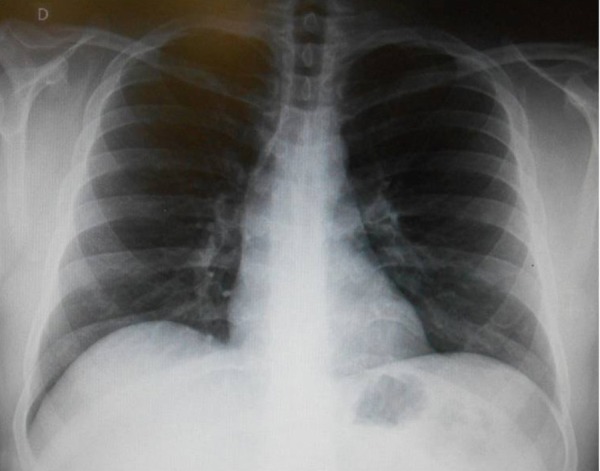
Chest radiograph shows normal heart area and pulmonary vascular bed. The elongated, dilated right upper arch may result from the modest increase of ascending aorta.

**Chest radiography** showed normal heart area and pulmonary vascular bed (Figure 1).

**Echocardiography** showed no contractile or morphological changes, normal-sized cardiac chambers and normal ventricular function (71%).

**Figure 2:**
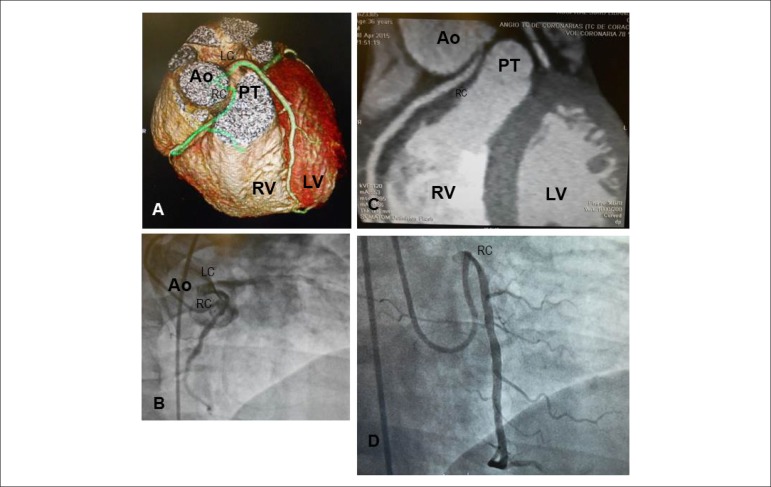
Similar projections of coronary arteries by computed tomography angiography (A, B) and cardiac catheterization (C, D) highlight the origin of the right coronary artery in the left sinus of Valsalva, the interarterial course and sharp narrowing in its beginning. AO: aorta; RC: right coronary artery; LC: left coronary artery; RV: right ventricle; LV: left ventricle; PT: pulmonary trunk.

**Coronary Computed Tomography Angiography** revealed anomalous origin of the right coronary artery from the left Valsalva sinus, with interarterial course between aorta and pulmonary trunk, with distinct narrowing of its proximal third and coronary ostium. The interarterial course of the right coronary artery was estimated at 10 mm (Figure 2).

**Cardiac catheterization** revealed the same aspect of the dominant right coronary artery, with anomalous origin from the left sinus of Valsalva and proximal course with sharp angulation and no obstructions (Figure 2). Left anterior descending artery and circumflex artery originate from the bifurcated left coronary artery; long diagonal and septal branches; absence of collateral circulation or arterial obstruction.

**TcMIBI stress myocardial scintigraphy** did not show ischemia, but indicated pain in the left sternal border, of low intensity and minutes of duration. Images obtained after isotonic exercise and at rest showed normal perfusion of the left ventricular walls.

Functional images combined with ECG (GSPECT) indicate normal motion and thickness of the left ventricular wall, and normal ejection fraction of the left ventricle (65%).

**Clinical Diagnosis:** Anomalous origin of the right coronary artery from the left Valsalva sinus, with interarterial course between the ascending aorta and pulmonary trunk, with signs and symptoms of arterial obstruction, but no evidence of myocardial ischemia.

**Clinical reasoning:** symptoms of low cardiac output combined with chest pain (even non-specific) suggest, a priori, aortic valve or coronary arterial malformation. The former was rejected due to the absence of heart murmurs. The abnormal origin of right coronary artery in the left sinus of Valsalva and its compression in the interarterial course were demonstrated by tomography.

**Differential diagnosis:** the same clinical picture may be seen in other coronary abnormalities, including the anomalous origin of the left coronary artery from the pulmonary trunk, but with large collateral circulation from the right coronary, enabling the development until adult age, in addition to the left coronary artery originating from the right sinus of Valsalva, with significant interarterial compression.

**Management:** The indication of surgery was evident in light of the clinical presentation of low cardiac output, chest pain (even non-specific), and unfavorable anatomy of the right coronary artery with interarterial compression, due to the risk of sudden death and/or ventricular arrhythmias, although no evidence of ischemia was detected by myocardial scintigraphy.

During the heart surgery, dissection of the right coronary artery originating from next to the left coronary ostium in the left sinus of Valsalva with an interarterial course between the aorta and pulmonary trunk was performed. The right coronary artery exhibited non-obstructive plaques in its proximal third, where it was cut and anastomosed to the anterior wall of the aorta with continuous suture (7-0 Prolene).

**Comments:** Anomalous origin of the right or left coronary artery from the sinus of Valsalva is a rare, congenital abnormality, and most of patients are asymptomatic. However, despite this apparently favorable condition, identified during routine or symptom assessment, the indication of surgery should be considered in order to prevent sudden death. This is a literature consensus, and few authors adopt a different viewpoint due to a conservative attitude. This approach may be used in the absence of symptoms or myocardial ischemia. Right coronary artery anomalies are ten times more common than in the left coronary artery. Physical effort is the trigger of ischemic symptoms, often fatal and without notice, be it for the interarterial obstruction, be it for the coronary artery arising from the aorta at an acute angle. The necessity of surgery is based on symptoms, regardless of the negative result for ischemia. The currently most accepted surgical techniques recommend reimplantation of the coronary artery in the sinus of Valsalva. Both coronary artery bypass surgery and the internal thoracic artery bypass have not been the approaches of choice due to the possibility of future obstructions, similarly to the placement of stents, which would continue to be exposed to the action of the arteries.^1-3^
